# Anticancer effects of a non-narcotic opium alkaloid medicine, papaverine, in human glioblastoma cells

**DOI:** 10.1371/journal.pone.0216358

**Published:** 2019-05-17

**Authors:** Mana Inada, Mika Shindo, Kyousuke Kobayashi, Akira Sato, Yohei Yamamoto, Yasuharu Akasaki, Koichi Ichimura, Sei-ichi Tanuma

**Affiliations:** 1 Department of Biochemistry, Faculty of Pharmaceutical Sciences, Tokyo University of Science, Noda, Chiba, Japan; 2 National Cancer Center Hospital, Chuo-ku, Tokyo, Japan; 3 Department of Neurosurgery, Jikei University School of Medicine, Minato-ku, Tokyo, Japan; 4 Division of Brain Tumor Translational Research, National Cancer Center Research Institute, Chuo-ku, Tokyo, Japan; 5 Department of Genomic Medicinal Science, Research Institute for Science and Technology, Organization for Research Advancement, Tokyo University of Science, Noda, Chiba, Japan; Sechenov First Medical University, RUSSIAN FEDERATION

## Abstract

The interaction between high-mobility group box 1 protein (HMGB1) and receptor for advanced glycation end products (RAGE) is important for tumor cell growth. We investigated the tumor biological effects of HMGB1 and RAGE interaction. Previously, we identified an inhibitor of HMGB1/RAGE interaction, papaverine (a non-narcotic opium alkaloid), using a unique drug design system and drug repositioning approach. In the present study, we examined the anticancer effects of papaverine in human glioblastoma (GBM) temozolomide (TMZ; as a first-line anticancer medicine)-sensitive U87MG and TMZ-resistant T98G cells. HMGB1 supplementation in the culture medium promoted tumor cell growth in T98G cells, and this effect was canceled by papaverine. In addition, papaverine in T98G cells suppressed cancer cell migration. As an HMGB1/RAGE inhibitor, papaverine also significantly inhibited cell proliferation in U87MG and T98G cells. The effects of papaverine were evaluated *in vivo* in a U87MG xenograft mouse model by determining tumor growth delay. The results indicate that papaverine, a smooth muscle relaxant, is a potential anticancer drug that may be useful in GBM chemotherapy.

## Introduction

High-mobility group box 1 (HMGB1) is a nonhistone DNA-binding nuclear protein that functions as an extracellular signaling molecule during inflammation, cell differentiation, cell migration, and tumor metastasis [1–4]. HMGB1 associates with high affinity to several receptors, including receptor for advanced glycation end products (RAGE) and Toll-like receptors (e.g., TLR-2, TLR-4, and TLR-9) [1–4]. RAGE is a multiligand receptor that binds structurally diverse molecules including HMGB1, S100 family members, and amyloid-β [1–4]. Its activation has been implicated in inflammation, tumor cell growth, migration, and invasion [1–4]. We have been investigating the relationship between the growth and migration of cancer cells and HMGB1/RAGE interaction in tumors, and recently we demonstrated that papaverine inhibits RAGE-dependent nuclear factor-κB activation, which is triggered by the RAGE ligand HMGB1 [5]. In addition, papaverine suppressed RAGE-dependent cell proliferation, migration, and cell invasion in human fibrosarcoma HT1080 cells [5]. We also previously reported a unique *in silico* drug design system [6]. Using a combination of this drug design system and a drug repositioning approach, we identified papaverine as an inhibitor of HMGB1/RAGE interaction [7].

Papaverine, a non-narcotic opium alkaloid, is isolated from *Papaver somniferum* [8]. Medicinal papaverine is used as a smooth muscle relaxant for the treatment of vasospasm and erectile dysfunction and functions by inhibiting phosphodiesterase 10A [9–11]. In cancer research, papaverine showed selective anticancer effects in several tumor cells, including prostate carcinoma LNCaP [12, 13] and PC-3 [14]; colorectal carcinoma HT29 [15]; breast carcinoma T47D [15], MCF-7, and MDA-MB-231 [16]; fibrosarcoma HT1080 [15]; and hepatocarcinoma HepG2 [17]. Benej *et al*. reported that papaverine radiosensitizes lung A549 and breast EO771 tumor cells by targeting mitochondrial complex 1 [18].

Glioblastoma (GBM) is the most aggressive primary malignant brain tumor with a median overall survival of 15 months [19–21]. Conventional treatments for patients with newly diagnosed GBM include surgery, radiotherapy, and temozolomide (TMZ) chemotherapy. TMZ is an alkylating agent prodrug that transmits a methyl group to the purine bases of DNA, i.e., *O*^6^-guanine, *N*^7^-guanine, and *N*^3^-adenine. However, *O*^6^-methylguanine-DNA methyltransferase (MGMT) directly repairs the main cytotoxic lesion caused by TMZ-mediated *O*^6^-guanine methylation, which could be the main mechanism of TMZ resistance [21]. Mismatch repair and base excision repair further contribute to TMZ resistance [21]. Therefore, the discovery of novel anticancer drugs is important for GBM chemotherapy.

In the present study, we assessed the anticancer effects of papaverine in human GBM U87MG and T98G cells as well as a U87MG xenograft mouse model. Papaverine significantly inhibited cell proliferation in U87MG and T98G cells and tumor growth in the U87MG xenograft mouse model. These observations suggest that the HMGB1/RAGE inhibitor papaverine can provide a novel anticancer strategy for GBM.

## Materials and methods

### Reagents

Papaverine hydrochloride was obtained from FUJIFILM Wako Pure Chemical Corporation (Osaka, Japan). Papaverine was stored as a 30 mM stock solution in ultrapure water at −20°C. TMZ was obtained from LKT Laboratories, Inc. (St. Paul, MN, USA) and stored as a 150 mM stock solution in dimethyl sulfoxide (Sigma-Aldrich; Merck KGaA, Darmstadt, Germany) at −20°C. Bovine native HMGB1 was obtained from Chondrex, Inc. (Redmond, WA, USA).

### Cell culture

Human GBM U87MG and T98G cell lines were obtained from the American Type Culture Collection (Manassas, VA, USA). U87MG and T98G cell lines were cultured in E-MEM and RPMI-1640, respectively, containing 10% heat-inactivated fetal bovine serum, 100 units/mL penicillin, and 100 μg/mL streptomycin. Cells were maintained in an incubator at 37°C with 5% CO_2_ at 100% relative humidity.

### Immunoblot analysis

Immunoblot analysis was performed as previously described [22, 23]. The following antibodies were used: anti-MGMT (1:1,000; Cell Signaling Technology, Danvers, MA, USA), anti-RAGE (1:1,000; Cell Signaling Technology, Tokyo, Japan), anti-glyceraldehyde 3-phosphate dehydrogenase (GAPDH; 1:20,000; Trevigen, Gaithersburg, MD, USA), horseradish peroxidase-linked anti-rabbit IgG (1:20,000; GE Healthcare UK Ltd., Amersham Place, Little Chalfont, UK), and horseradish peroxidase-linked whole antibody anti-mouse IgG (1:20,000; GE Healthcare).

### Trypan blue dye exclusion assay

Cell viability was calculated using the TC20 automated cell count system (Bio-Rad, Hercules, CA, USA) as the number of live cells divided by the total number of cells on a cell count slide.

### Cell migration assay

A cell migration assay was performed as previously described [5]. T98G cells were passed onto 3.5 cm dishes (2.0 × 10^5^ cells per dish) and cultured to about 100% confluence in each dish. Then, cells were wounded by denuding a strip of the monolayer with a 1,000 μL pipette tip. Cells were washed with Gibco Opti-MEM medium (Life Technologies Limited, Paisley, UK) and then incubated for 24 h at 37°C under humidified 5% CO_2_ in Opti-MEM medium with 0 (water alone; vehicle), 100, or 300 μM papaverine. The wound area was photographed 0 h and 24 h after scratching using a Leica DMi1 light microscope with a 5 × objective (Ernst-Leitz-strasse, Wetzlar, Germany). The pictures were analyzed by the image processing program Image J. The ratio of cell migration (%) was calculated as follows: (wound distance at 0 h—wound distance at 24 h)/wound distance at 0 h × 100.

### Cell activity WST-8 assay

A cell activity WST-8 assay was performed as previously described [23]. Cells were briefly passed onto 96-well plates (1,000 cells per well) in triplicates and then treated with various concentrations of drugs or ultrapure water (as a negative control). After incubation for 72 h, 20 μL WST-8 reagent was added to each well and the plate was placed in a 5% CO_2_ incubator at 37°C for an additional 1 h. Optical density was measured at 490 nm on a Tecan microplate reader (Männedorf, Switzerland).

### Human GBM U87MG xenograft mouse model

All animal studies were approved by the Animal Experimental Committee of Tokyo University of Science (TUS) and performed in accordance with the Guidelines for Animal Experiments of the TUS, which meet the ethical guidelines for experimental animals in Japan. The animals were kept at 23 ± 2 °C under specific pathogen-free conditions with a 12 h light/dark cycle and had free access to a standard diet and water. For heterotrophic/subcutaneous xenografts, 1 × 10^6^ U87MG cells resuspended in 100 μL phosphate-buffered saline (PBS) were subcutaneously injected into the right leg of 5-week-old male BALB/c nude mice (CLEA Japan, Inc., Tokyo, Japan) weighing approximately 20–22 g with four mice in each group. Before tumor inoculation, mice were anesthetized with isoflurane (Escain^®^ inhalation anesthesia liquid 1 mL/mL, Pfizer Inc, NY, USA). Papaverine was diluted in saline. After 11–13 d of inoculation, papaverine (40 mg/kg) or saline (vehicle control, solvent alone) was intraperitoneally administrated to four mice per group twice a day for 4 d. Tumor size was monitored once every 3–4 d. Tumor volume (*V*) was calculated using the following formula: *V* = *ab*^2^/2 (*a* and *b* are the long and short diameters of the tumor, respectively). The protocol was approved by the Committee (Y16034 and Y15052). Mice were sacrificed by isoflurane inhalation followed by cervical dislocation. In the animal experiments, humane endpoint criteria were defined as tumor burden > 10% of body weight, tumor volume > 2,000 mm^3^, or tumor largest dimension > 20 mm.

### Statistical analysis

Data are presented as the mean ± SE. The significance of the differences among groups was evaluated using the Student’s *t*-test. *P* < 0.05 was considered to be statistically significant.

## Results and discussion

### HMGB1 promoted cancer cell proliferation in human GBM U87MG and T98G cells

**Fig 1:**
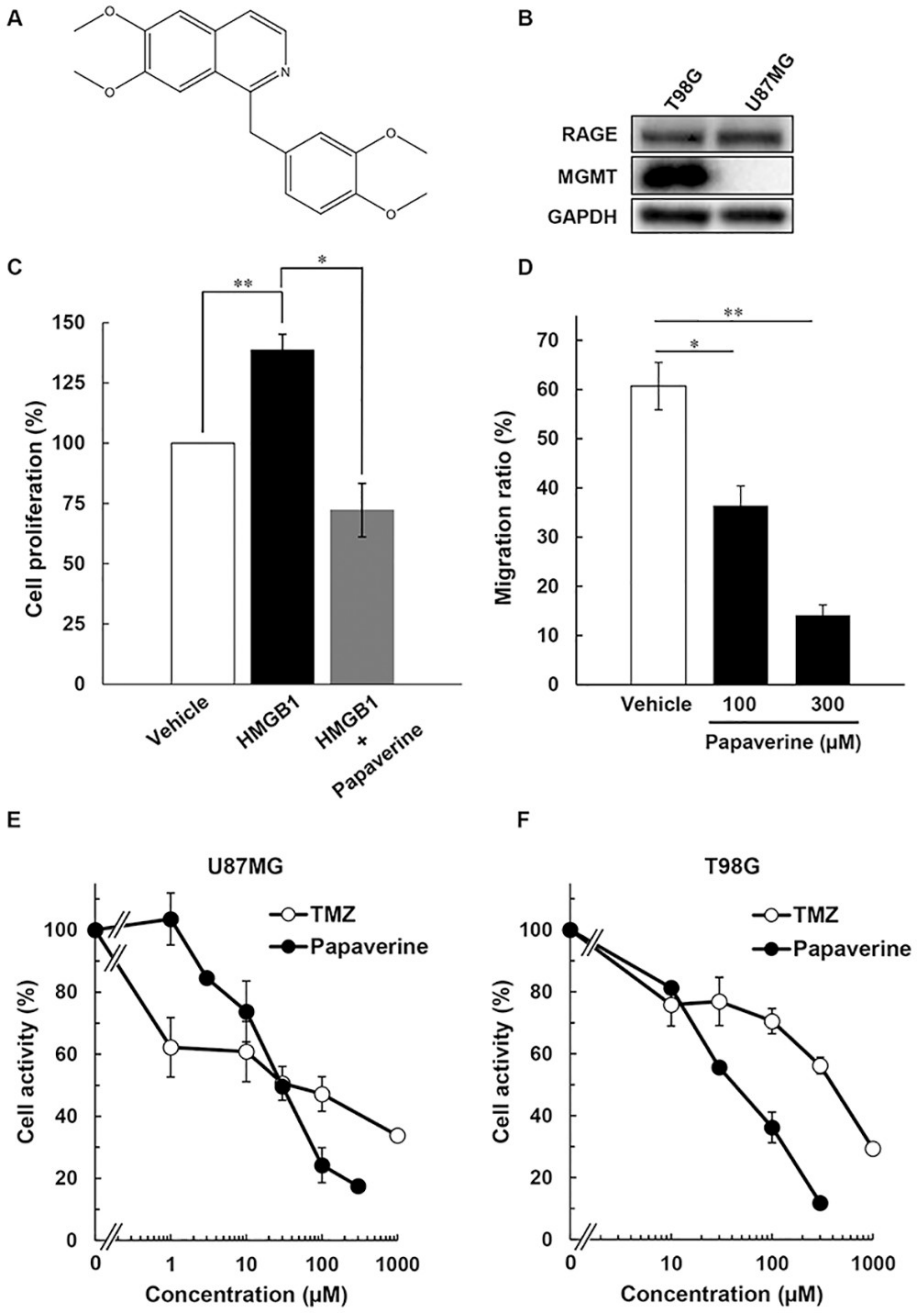
Antiproliferative activity of papaverine in human GBM U87MG and T98G cells. (A) Chemical structure of papaverine. (B) Protein levels of MGMT, RAGE, and GAPDH analyzed by immunoblotting. GAPDH used as an internal control. Data are representative of at least three independent experiments. (C) T98G cells treated with 10 μg/mL bovine HMGB1 protein or vehicle (PBS) and then incubated for 72 h. Cells were counted by trypan blue dye exclusion assay using the TC20 automated cell count system. Cell proliferation (%) represents the mean ± SE of three independent experiments. *P* values were calculated against vehicle control with the Student’s *t*-test. **p < 0.05, **p < 0.01. (D) The migration ability of T98G cells was analyzed in an *in vitro* scratch assay. T98G cells were treated with papaverine at the indicated concentration or water as a vehicle for 24 h. The migration ratio (%) represents the mean ± SE of triplicate experiments. Similar results were obtained in two independent experiments. *P* values were calculated against vehicle control with the Student’s *t*-test. *p < 0.05, **p < 0.01. (E) U87MG and (F) T98G cells were examined for cell activity in a WST-8 assay after 72 h papaverine treatment. Results are the averages of three independent experiments with error bars showing SE from triplicates.

We studied the association between cell proliferation and HMGB1/RAGE interaction in several tumor cells. Using an *in silico* drug design system and a drug repositioning approach, we found that a non-narcotic opium alkaloid, papaverine (Fig 1A), inhibits HMGB1/RAGE interaction [7]. Herein, we investigated the anticancer effects of papaverine in human GBM MGMT-negative/TMZ-sensitive U87MG and MGMT-positive/TMZ-resistant T98G cells. First, we analyzed the protein levels of our drug target, RAGE, and the TMZ-resistant marker MGMT in these cells by immunoblotting. As shown in Fig 1B (top panel), RAGE protein levels were almost identical in these cells. Conversely, MGMT expression was higher in T98G but not detected in U87MG cells (Fig 1B, middle panel). To examine the response of HMGB1 to cancer cell proliferation, we treated T98G cells with supplemental 10 μg/mL HMGB1. It is known that supplementation of 10 μg/mL HMGB1 promotes cell proliferation in human GBM U87MG and T98G cells (S1 Table). Proliferation in T98G cells significantly increased (by approximately 40%) upon HMGB1 treatment (Fig 1C and S1 Table). However, papaverine inhibited HMGB1-promoted cell proliferation. In addition, papaverine in T98G cells suppressed cancer cell migration in a dose-dependent manner (Fig 1D). Bassi *et al*. previously reported that HMGB1 promotes cancer cell growth and migration by acting as an autocrine factor in human GBM T98G cells [24]. These findings suggest that the inhibition of the HMGB1/RAGE interaction may be highly effective in GBM chemotherapy.

### Papaverine inhibited cancer cell proliferation in human GBM U87MG and T98G cells

We studied the anticancer effects of papaverine in U87MG and TMZ-resistant T98G cells with a WST-8 assay. The EC_50_ for papaverine was 29 and 40 μM in U87MG and T98G cells, respectively (Table 1, Fig 1E and 1F). Conversely, the EC_50_ of the current primary anticancer agent TMZ was 42 and 390 μM in U87MG and T98G cells, respectively (Table 1, Fig 1E and 1F). These data indicate that papaverine is effective in both human GBM U87MG and TMZ-resistant T98G cells. Xue *et al*. previously reported that papaverine induces the reversible opening of the blood–brain barrier (BBB) [25]. Further, Bhattacharjee *et al*. reported that papaverine mediates transient BBB permeability [26]. These findings suggest that papaverine is a potential therapeutic agent for TMZ-resistant GBM.

**Table 1:** Summary of the anticancer effects of papaverine in human GBM U87MG and T98G cells.

	EC_50_ (μM; WST-8, 72 h)	
	U87MG	T98G
**Papaverine**	29	40
**TMZ**	42	390

Cells were treated as described in Fig 1. EC_50_ values are the averages of triplicate determinations obtained in at least three independent experiments.

Interestingly, Qi and coworkers generated TMZ-resistant U87MG cells from U87MG cells by treatment with TMZ for 6 months [27]. It is important to compare the anticancer effects of papaverine in between TMZ-resistant and TMZ-sensitive U87MG cell lines with similar genetic backgrounds. We are currently generating TMZ-resistant U87MG cells from parent U87MG cells. In future, we would like to further investigating the anticancer effects and mechanisms of papaverine in TMZ-resistant U87MG cells and TMZ-sensitive U87MG cells (parent cell line).

### Papaverine suppressed tumor cell growth in a U87MG xenograft mouse model

**Fig 2:**
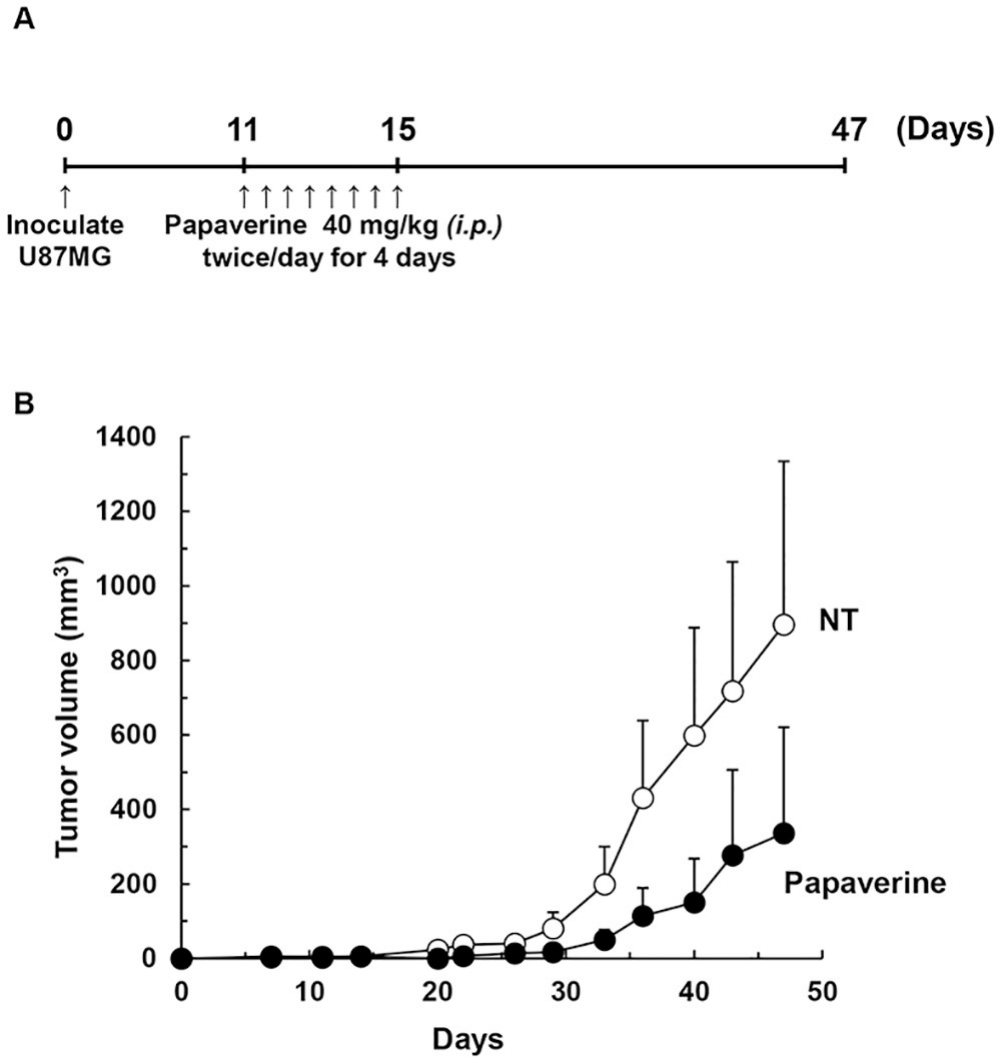
Anticancer effects of papaverine in a human GBM U87MG xenograft mouse model. (A) Experimental schedule. (B) To assess the effect of papaverine in tumors in a xenograft model, 1 × 10^6^ U87MG human GBM cells were subcutaneously injected into the right leg of 5-week-old male BALB/c nude mice. After 11–13 d of inoculation, four mice per group were treated with papaverine (40 mg/kg) or saline (vehicle control, solvent alone) twice a day for 4 d via i.p. administration. Tumor size was measured once every 3–4 d. Tumor volume (*V*) was calculated as described in Materials and methods. Results are the averages for groups of four mice each with error bars showing SE. White circle, control; black circle, papaverine.

We investigated the antitumor activity of papaverine in a U87MG xenograft mouse model (Fig 2A). The effects of papaverine on tumor volume were monitored for 36 d after treatment with papaverine. In this xenograft model, the tumor volume on day 47 was reduced by approximately 63% with papaverine treatment compared to the vehicle control (tumor volume, mean ± SE; 336 ± 285 and 896 ± 438 mm^3^, respectively; Table 2 and Fig 2B). This result indicates that papaverine has strong antitumor activity in this xenograft model. In addition, the final tumor volume (mean ± SE) on day 54 was 642 ± 545 mm^3^ in papaverine-treated mice (Table 2 and S2 Table). This finding suggests that papaverine delays tumor growth. Papaverine has been found to selectively inhibit cancer cell proliferation in several solid tumors (i.e., prostate, colorectal, breast, and lung cancer as well as hepatocarcinoma and fibrosarcoma) [12–18]. Additionally, several research groups have reported that papaverine promotes transient BBB permeability [25, 26]. Papaverine is also a vasodilator used for the prevention of intraoperative vasospasm during craniotomy (e.g., subarachnoid hemorrhage) [28–30]. These reports and our novel findings suggest that papaverine may be effective against human GBM.

**Table 2:** Summary of the anticancer effects of papaverine in a human GBM U87MG xenograft mouse model.

Days	Tumor volume (mm^3^)			
	33	40	47	54
**Saline**	199 ± 101	598 ± 290	896 ± 438	-
**Papaverine**	50 ± 27	150 ± 118	336 ± 285	642 ± 545

Tumor volume was calculated as described in Materials and methods. Results are the means ± SE for groups of four mice.

## Conclusions

HMGB1 promotes cancer cell proliferation in human GBM U87MG and T98G cells. Additionally, papaverine inhibits cancer cell proliferation in human GBM TMZ-sensitive U87MG and TMZ-resistant T98G cells. Moreover, papaverine dramatically suppresses tumor volume in a human GBM U87MG xenograft mouse model. In the future, we will attempt to perform clinical trials to evaluate the anticancer effects and safety of papaverine.

## Supporting information

S1 Table HMGB1 induces cell proliferation in human GBM U87MG and T98G cells. Cells were treated with 10 μg/mL bovine HMGB1 protein or vehicle (PBS) and then incubated for 72 h. Cells counted by trypan blue dye exclusion assay using TC20 automated cell count system. Cell proliferation (%) represents the average of three independent experiments.
(PDF)

S2 Table Summary of the anticancer effects of papaverine in a human GBM U87MG xenograft mouse model. Tumor volume was calculated as described in Materials and methods. Results are the means ± SE for groups of four mice.
(PDF)

## Abbreviations

BBB: blood–brain barrier
GBM: glioblastoma
HMGB1: high-mobility group box 1 protein
MGMT: *O*^6^-methylguanine-DNA methyltransferase
PBS: phosphate-buffered saline
RAGE: receptor for advanced glycation end products
TMZ: temozolomide

## References

[1] Sims GP, Rowe DC, Rietdijk ST, Herbst R, Coyle AJ. HMGB1 and RAGE in inflammation and cancer. Annual Review of Immunology. 2010;28:367–88. Epub 2010/03/03. 10.1146/annurev.immunol.021908.132603 . 20192808

[2] Tang D, Kang R, Zeh HJ 3rd, Lotze MT. High-mobility group box 1 and cancer. Biochimica et Biophysica Acta. 2010;1799(1–2):131–40. Epub 2010/02/04. 10.1016/j.bbagrm.2009.11.014 . 20123075 PMC2818552

[3] Wu L, Yang L. The function and mechanism of HMGB1 in lung cancer and its potential therapeutic implications. Oncology Letters. 2018;15(5):6799–805. Epub 2018/05/05. 10.3892/ol.2018.8215 . 29725415 PMC5920474

[4] He SJ, Cheng J, Feng X, Yu Y, Tian L, Huang Q. The dual role and therapeutic potential of high-mobility group box 1 in cancer. Oncotarget. 2017;8(38):64534–50. Epub 2017/10/04. 10.18632/oncotarget.17885 . 28969092 PMC5610024

[5] El-Far A, Munesue S, Harashima A, Sato A, Shindo M, Nakajima S, et al In vitro anticancer effects of a RAGE inhibitor discovered using a structure-based drug design system. Oncology Letters. 2018;15(4):4627–34. Epub 2018/03/16. 10.3892/ol.2018.7902 . 29541234 PMC5835888

[6] Sakai J, Yoshimori A, Nose Y, Mizoroki A, Okita N, Takasawa R, et al Structure-based discovery of a novel non-peptidic small molecular inhibitor of caspase-3. Bioorganic & Medicinal Chemistry. 2008;16(9):4854–9. Epub 2008/04/05. 10.1016/j.bmc.2008.03.046 . 18387304

[7] Tamada K, Nakajima S, Ogawa N, Inada M, Shibasaki H, Sato A, et al Papaverine identified as an inhibitor of high mobility group box 1/receptor for advanced glycation end-products interaction suppresses high mobility group box 1-mediated inflammatory responses. Biochemical and Biophysical Research Communications. 2019;511(3):665–70. Epub 2019/03/04. 10.1016/j.bbrc.2019.01.136 . 30826057

[8] Merck G. Vorläufige Notiz über eine neue organische Base im Opium. Justus Liebigs Annalen der Chemie. 1848;66(1):125–8.

[9] Liu HM, Tu YK. The efficacy of papaverine administration by different routes for the treatment of experimental acute cerebral vasospasm. Journal of Clinical Neuroscience: official journal of the Neurosurgical Society of Australasia. 2002;9(5):561–5. Epub 2002/10/18. . 12383416 10.1054/jocn.2001.1036

[10] Chappie TA, Humphrey JM, Allen MP, Estep KG, Fox CB, Lebel LA, et al Discovery of a series of 6,7-dimethoxy-4-pyrrolidylquinazoline PDE10A inhibitors. Journal of Medicinal Chemistry. 2007;50(2):182–5. Epub 2007/01/19. 10.1021/jm060653b . 17228859

[11] Yildiz N, Gokkaya NK, Koseoglu F, Gokkaya S, Comert D. Efficacies of papaverine and sildenafil in the treatment of erectile dysfunction in early-stage paraplegic men. International Journal of Rehabilitation Research Internationale Zeitschrift fur Rehabilitationsforschung Revue internationale de recherches de readaptation. 2011;34(1):44–52. Epub 2010/08/12. 10.1097/MRR.0b013e32833d6cb2 . 20700057

[12] Goto T, Matsushima H, Kasuya Y, Hosaka Y, Kitamura T, Kawabe K, et al The effect of papaverine on morphologic differentiation, proliferation and invasive potential of human prostatic cancer LNCaP cells. International Journal of Urology: official journal of the Japanese Urological Association. 1999;6(6):314–9. Epub 1999/07/15. . 10404308 10.1046/j.1442-2042.1999.00069.x

[13] Shimizu T, Ohta Y, Ozawa H, Matsushima H, Takeda K. Papaverine combined with prostaglandin E2 synergistically induces neuron-like morphological changes and decrease of malignancy in human prostatic cancer LNCaP cells. Anticancer Research. 2000;20(2a):761–7. Epub 2000/05/16. . 10810351

[14] Huang H, Li LJ, Zhang HB, Wei AY. Papaverine selectively inhibits human prostate cancer cell (PC-3) growth by inducing mitochondrial mediated apoptosis, cell cycle arrest and downregulation of NF-kappaB/PI3K/Akt signalling pathway. Journal of BUON: official journal of the Balkan Union of Oncology. 2017;22(1):112–8. Epub 2017/04/04. . 28365943

[15] Afzali M, Ghaeli P, Khanavi M, Parsa M, Montazeri H, Ghahremani MH, et al Non-addictive opium alkaloids selectively induce apoptosis in cancer cells compared to normal cells. Daru: Journal of Faculty of Pharmacy, Tehran University of Medical Sciences. 2015;23:16 Epub 2015/04/19. 10.1186/s40199-015-0101-1 . 25890335 PMC4341877

[16] Sajadian S, Vatankhah M, Majdzadeh M, Kouhsari SM, Ghahremani MH, Ostad SN. Cell cycle arrest and apoptogenic properties of opium alkaloids noscapine and papaverine on breast cancer stem cells. Toxicology Mechanisms and Methods. 2015;25(5):388–95. Epub 2015/05/20. 10.3109/15376516.2015.1045656 . 25980655

[17] Noureini SK, Wink M. Antiproliferative effect of the isoquinoline alkaloid papaverine in hepatocarcinoma HepG-2 cells—inhibition of telomerase and induction of senescence. Molecules (Basel, Switzerland). 2014;19(8):11846–59. Epub 2014/08/12. 10.3390/molecules190811846 PMC6271551 25111025

[18] Benej M, Hong X, Vibhute S, Scott S, Wu J, Graves E, et al Papaverine and its derivatives radiosensitize solid tumors by inhibiting mitochondrial metabolism. Proceedings of the National Academy of Sciences of the United States of America. 2018;115(42):10756–61. Epub 2018/09/12. 10.1073/pnas.1808945115 . 30201710 PMC6196495

[19] Laperriere N, Zuraw L, Cairncross G. Radiotherapy for newly diagnosed malignant glioma in adults: a systematic review. Radiotherapy and Oncology: Journal of the European Society for Therapeutic Radiology and Oncology. 2002;64(3):259–73. Epub 2002/09/21. . 12242114 10.1016/s0167-8140(02)00078-6

[20] Huse JT, Holland EC. Targeting brain cancer: advances in the molecular pathology of malignant glioma and medulloblastoma. Nature Reviews Cancer. 2010;10(5):319–31. Epub 2010/04/24. 10.1038/nrc2818 . 20414201

[21] Zhang J, Stevens MF, Bradshaw TD. Temozolomide: mechanisms of action, repair and resistance. Current Molecular Pharmacology. 2012;5(1):102–14. Epub 2011/11/30. . 22122467 10.2174/1874467211205010102

[22] Sato A, Satake A, Hiramoto A, Wataya Y, Kim HS. Protein expression profiles of necrosis and apoptosis induced by 5-fluoro-2'-deoxyuridine in mouse cancer cells. Journal of Proteome Research. 2010;9(5):2329–38. Epub 2010/02/17. 10.1021/pr9010537 . 20155980

[23] Ogino Y, Sato A, Uchiumi F, Tanuma SI. Cross resistance to diverse anticancer nicotinamide phosphoribosyltransferase inhibitors induced by FK866 treatment. Oncotarget. 2018;9(23):16451–61. Epub 2018/04/18. 10.18632/oncotarget.24731 . 29662658 PMC5893253

[24] Bassi R, Giussani P, Anelli V, Colleoni T, Pedrazzi M, Patrone M, et al HMGB1 as an autocrine stimulus in human T98G glioblastoma cells: role in cell growth and migration. Journal of Neuro-oncology. 2008;87(1):23–33. Epub 2007/11/03. 10.1007/s11060-007-9488-y . 17975708

[25] Xue H, Wang H, Kong L, Zhou H. Opening blood-brain-barrier by intracarotid infusion of papaverine in treatment of malignant cerebral glioma. Chinese Medical Journal. 1998;111(8):751–3. Epub 2001/03/14. . 11245034

[26] Bhattacharjee AK, Kondoh T, Nagashima T, Ikeda M, Ehara K, Tamaki N. Quantitative analysis of papaverine-mediated blood-brain barrier disruption in rats. Biochemical and Biophysical Research Communications. 2001;289(2):548–52. Epub 2001/11/22. 10.1006/bbrc.2001.6029 . 11716508

[27] Yi GZ, Liu YW, Xiang W, Wang H, Chen ZY, Xie SD, et al Akt and beta-catenin contribute to TMZ resistance and EMT of MGMT negative malignant glioma cell line. Journal of the Neurological Sciences. 2016;367:101–6. Epub 2016/07/18. 10.1016/j.jns.2016.05.054 27423571

[28] Liu JK, Couldwell WT. Intra-arterial papaverine infusions for the treatment of cerebral vasospasm induced by aneurysmal subarachnoid hemorrhage. Neurocritical Care. 2005;2(2):124–32. Epub 2005/09/15. 10.1385/NCC:2:2:124 . 16159054

[29] Sayama CM, Liu JK, Couldwell WT. Update on endovascular therapies for cerebral vasospasm induced by aneurysmal subarachnoid hemorrhage. Neurosurgical Focus. 2006;21(3):E12 Epub 2006/10/13. . 17029336 10.3171/foc.2006.21.3.12

[30] Keyrouz SG, Diringer MN. Clinical review: Prevention and therapy of vasospasm in subarachnoid hemorrhage. Critical Care (London, England). 2007;11(4):220 Epub 2007/08/21. 10.1186/cc5958 . 17705883 PMC2206512

