# Supplementary material for: Anticancer effects of a non-narcotic opium alkaloid medicine, papaverine, in human glioblastoma cells
Source: PLoS One. 2019 May 17;14(5):e0216358. doi: 10.1371/journal.pone.0216358 (PMC6524804; doi:10.1371/journal.pone.0216358)
Supplement: S1 Table — Cells were treated with 10 μg/mL bovine HMGB1 protein or vehicle (PBS) and then incubated for 72 h. Cells counted by trypan blue dye exclusion assay using TC20 automated cell count system. Cell proliferation (%) represents the average of three independent experiments. (PDF) [file pone.0216358.s001.pdf]

S1 Table

	Cell proliferation (%, 72h)	
	**U87MG**	**T98G**
**Vehicle**	100	100
**HMGB1**	142	138
