# Supplementary material for: Anticancer effects of a non-narcotic opium alkaloid medicine, papaverine, in human glioblastoma cells
Source: PLoS One. 2019 May 17;14(5):e0216358. doi: 10.1371/journal.pone.0216358 (PMC6524804; doi:10.1371/journal.pone.0216358)
Supplement: S2 Table — Tumor volume was calculated as described in Materials and methods. Results are the means ± SE for groups of four mice. (PDF) [file pone.0216358.s002.pdf]

S2 Table

	Tumor volume (mm^3)													
**Days**	**7**	**11**	**14**	**20**	**22**	**26**	**29**	**33**	**36**	**40**	**43**	**47**	**50**	**54**
**Saline**	6 ± 5	5 ± 4	6 ± 5	24 ± 12	37 ± 18	40 ± 23	81 ± 43	199 ± 101	430 ± 209	598 ± 290	718 ± 347	896 ± 438	987 ± 473	-
**Papaverine**	4 ± 3	3 ± 1	6 ± 3	0 ± 0	6 ± 6	14 ± 7	17 ± 10	50 ± 27	114 ± 75	150 ± 118	277 ± 229	336 ± 285	466 ± 393	642 ± 545
